# Editorial: Novel approaches to targeting the vasculature and metabolome to prevent brain aging and related diseases

**DOI:** 10.3389/fncel.2024.1505939

**Published:** 2024-10-25

**Authors:** Jennifer Ihuoma, Sharon Negri, Amanda Morato Do Canto, Anika M. S. Hartz, Aditi Deshpande, Stefano Tarantini

**Affiliations:** ^1^Center for Geroscience and Healthy Brain Aging, Department of Biochemistry and Molecular Biology, University of Oklahoma Health Sciences Center, Oklahoma City, OK, United States; ^2^Peggy and Charles Stephenson Cancer Center, University of Oklahoma Health Sciences Center, Oklahoma City, OK, United States; ^3^Faculty of Medical Sciences, State University of Campinas, Campinas, Brazil; ^4^Sanders-Brown Center on Aging, College of Medicine, University of Kentucky, Lexington, KY, United States; ^5^Department of Pharmacology and Nutritional Sciences, College of Medicine, University of Kentucky, Lexington, KY, United States; ^6^BioAge Labs, Richmond, CA, United States; ^7^Hudson College of Public Health, University of Oklahoma Health Sciences Center, Oklahoma City, OK, United States

**Keywords:** neurovascular uncoupling, neurovascular unit, aging, neurodegenerative disease, neurometabolic alterations, blood brain barrier, brain homeostasis, cognitive decline

The brain, as the most metabolically active organ in the human body, consumes approximately 20% of the body's energy supply at rest. This high energy demand is fulfilled by processes like neurovascular coupling (NVC), which ensures that areas of the brain requiring more energy receive increased blood flow to supply oxygen and glucose. Neurovascular coupling is coordinated by the neurovascular unit (NVU), composed of neurons, glia, pericytes, endothelial cells, and smooth muscle cells. This unit also maintains the integrity of the blood-brain barrier (BBB), which regulates the exchange of molecules between the bloodstream and the brain. During brain development, the NVC is formed and ensures that energy requirements of the developing brain are met for fundamental processes such as synaptogenesis and neural plasticity. As we age, there is neurovascular and neurometabolic uncoupling and loss of BBB integrity, which leads to insufficient blood flow, impaired waste clearance, and bioenergetic deficits. Such disruptions have been linked to cognitive decline and neurodegenerative diseases such as Alzheimer's disease (AD), vascular cognitive impairment and dementia (VCID), and multiple sclerosis (MS) as well as metabolic conditions like hypercholesterolemia and insulin resistance. The goal of this Research Topic is to present cutting-edge basic and translational studies that shed light on the mechanisms driving NVU dysfunction and metabolic imbalance in the aging brain, as well as identify potential therapeutic strategies for preventing or mitigating cognitive decline and neurodegenerative disease (Maroto-Rodriguez et al., 2023; Dobreva et al., 2022; Balasubramanian et al., 2020; Bray et al., 2022). Below are six new perspectives and findings that address key aspects of this broad theme.

Adachi et al. contribute to the understanding of the NVU by focusing on perivascular macrophages (PVMs) and their role in amyotrophic lateral sclerosis (ALS) progression. The study shows that depletion of PVMs in an ALS mouse model improves blood-spinal cord barrier (BSCB) integrity and delays motor neuron loss. This aligns with the Research Topic's theme by emphasizing the importance of immune cells in maintaining BSCB function and neurovascular health. These results highlight new potential targets for therapeutic intervention in ALS and other neurodegenerative diseases where barrier dysfunction is a hallmark.

Expanding on the role of the NVU and immune cells in disease progression, Chen T. et al. identify neurotrophic factor receptor (NGFR) as a key biomarker associated with the disruption of NVC in diabetic macular edema (DME), highlighting its role in regulating inflammation and immunity through the neurotrophin signaling pathway. This work demonstrates how metabolic diseases such as diabetes can lead to neurovascular dysfunction resulting in vascular-related neurodegeneration. Moreover, authors suggest that identifying mediators of NVC may uncover therapeutic targets for early intervention in DME.

In a similar vein, Fraga et al. uncover mechanisms underlying another vascular-related neurodegenerative condition, VCID. By examining the effects of chronic cerebral hypoperfusion, often caused by carotid artery stenosis, on hippocampal neurogenesis in mice, the study reveals that impaired neurogenesis and increased cell death contribute to spatial memory deficits. The findings exemplify how disruptions in NVC leading to reduced cerebral blood flow and loss of BBB integrity can accelerate VCID and cognitive decline, making it a critical addition to the discussion on preventing age-related cognitive diseases through vascular interventions.

Further elaborating on the role of the BBB in neurodegenerative diseases, Ucar et al. focus on the integral NVU member, pericytes, in the pathophysiology of MS, a disease marked by neuroinflammation and BBB dysfunction. Pericytes maintain the BBB, respond to neuroinflammation, differentiate to myofibroblasts and mediate fibrosis in MS. Treatment with Carbenoxolone, a hemichannel blocker, significantly reduced fibrosis in pericytes, thereby mitigating disease progression in a mouse model of MS. This study highlights pericytes as contributors to MS progression, thus aligning with the broader theme of cerebrovascular health by focusing on how fibrosis and BBB dysfunction contribute to neurodegenerative diseases. By showing that pericytes are pivotal in the accumulation of fibrotic extracellular matrix in MS, Ucar et al. open the field to studying anti-fibrotic therapeutic avenues to preserve the NVU and delay progressive neurodegeneration in MS.

Maintaining cerebrovascular health is not just neuroprotective in the aging brain but is also vital during neurodevelopment, as demonstrated by Chen Y-C. et al. Their study, using a zebrafish model, uncovers the dual role of angiopoietin 1 (angpt1) and integrin beta 1b (itgb1b) in regulating both, cerebrovascular development and neurogenesis. Using gain-of-function and loss-of-function mutations, they identify how angpt1 and itgb1b are critical for cerebrovascular formation and exert their neurogenic effects through Notch and Wnt signaling pathways, influencing the patterning of the developing brain. This is particularly relevant to the NVC since proper vascular development is essential for maintaining the brain's metabolic demands. Moreover, this study offers insights into how neurodevelopmental processes might be exploited therapeutically in neurodegenerative conditions where angiogenesis and neural proliferation are disrupted.

Finally, Trinh et al. review the role of SIRT3, a mitochondrial protein deacetylase, in maintaining cellular energy homeostasis—a critical factor for brain health. SIRT3 is a key player in mitochondrial function, regulating reactive oxygen species (ROS), ATP production, and mitochondrial dynamics, all of which are vital for sustaining the NVU and neurometabolic functions. With focus on age-related decline in mitochondrial efficiency linked to cognitive impairment and neurovascular dysfunction, this review connects the regulation of SIRT3 with potential interventions for neurovascular aging. By maintaining cellular homeostasis, SIRT3 offers a promising therapeutic target to counteract the oxidative stress and metabolic disruptions observed in neurodegenerative diseases like AD, where neurovascular uncoupling is prevalent.

In conclusion, the research presented here highlights the complex interplay between neurovascular health, BBB integrity, and metabolic regulation, all of which are essential for maintaining brain homeostasis throughout life. As the brain ages, impairments in these systems contribute to cognitive decline and neurodegenerative disease. Each study uniquely identifies mechanisms that provide a better understanding of how disruptions in the NVU and metabolic processes accelerate neurodegeneration. Collectively, the studies underscore the importance of targeting both cerebrovascular and metabolic pathways to preserve brain function, from mitochondrial regulators like SIRT3 to pericytes, PVMs, and novel biomarkers such as NGFR. As the field advances, the integration of these molecular and cellular insights will be crucial for developing interventions that protect the aging brain from the ravages of neurovascular uncoupling and metabolic imbalance.
